# Rewiring redox defense: Nrf2-driven antioxidant signaling as a gateway to programmed cell death activation in colorectal cancer

**DOI:** 10.1016/j.gendis.2026.102228

**Published:** 2026-05-08

**Authors:** Yichen Yu, Bin Ma, Yue Liu, Ziyi Wang, Jian Chen, Xiaoxi Li, Shulan Sun, Simeng Bao

**Affiliations:** a Cancer Research Institute, Central Laboratory, Cancer Hospital of China Medical University, Liaoning Cancer Hospital & Institute, Shenyang, Liaoning 110042, China; b Key Laboratory of Environmental Stress and Chronic Disease Control and Prevention, Ministry of Education, China Medical University, Shenyang, Liaoning 110122, China; c Key Laboratory of Liaoning Province on Toxic and Biological Effects of Arsenic, China Medical University, Shenyang, Liaoning 110122, China; d Department of Colorectal Surgery, Cancer Hospital of China Medical University, Liaoning Cancer Hospital & Institute, Shenyang, Liaoning 110042, China; e Department of Endocrinology and Metabolism, Institute of Endocrinology, Liaoning Provincial Key Laboratory of Endocrine Diseases, The First Affiliated Hospital of China Medical University, Shenyang, Liaoning 110001, China

**Keywords:** Apoptosis, Autophagy, Colorectal cancer, Combination therapy, Ferroptosis, Nrf2 activity, Oxidative stress

## Abstract

Nuclear factor erythroid 2-related factor 2 (Nrf2), a master regulator of antioxidant and phase II detoxification genes, plays a dual role in colorectal cancer (CRC) progression by maintaining redox homeostasis while paradoxically fostering chemoresistance. Programmed cell death (PCD) pathways, including apoptosis, ferroptosis, and autophagy, play a crucial role in the pathogenesis and therapeutic responsiveness of CRC, largely through mechanisms mediated by reactive oxygen species (ROS). Emerging evidence highlights that pharmacological agents or natural compounds targeting PCD trigger ROS accumulation, selectively impairing the survival of CRC cells due to their increased dependency on ROS. Although Nrf2’s dual roles have been extensively characterized, the specific interactions between Nrf2-mediated redox regulation and PCD modulation are not yet fully understood, thereby constraining the advancement of redox-based therapeutic strategies. This review synthesizes recent advances in Nrf2 activity modulation within PCD-centric CRC treatment paradigms, emphasizing its context-dependent roles in ROS management. Furthermore, we propose rational strategies to harness Nrf2 inhibitors or activators, either as monotherapies or in combination with conventional regimens, to overcome chemoresistance and amplify therapeutic efficacy. By bridging mechanistic insights with therapeutic potential, this work underscores Nrf2 as a pivotal node for redefining redox-targeted CRC interventions.

## Introduction

Colorectal cancer (CRC) represents one of the most prevalent malignant neoplasms within the gastrointestinal tract. It ranks as the second leading cause of cancer-related mortality among both men and women collectively.^1^ This condition has emerged as a critical public health issue, especially in light of the increasing incidence and mortality rates observed in younger adult populations.^2^^,^^3^ The pathogenesis of CRC is highly intricate, with genetic predispositions, environmental influences, and unhealthy lifestyle choices serving as significant risk factors for its development.^4^, ^5^, ^6^ The critical importance of early detection and intervention is underscored by the fact that CRC is frequently diagnosed at advanced stages, which are associated with less favorable prognoses. Consequently, elucidating the fundamental molecular mechanisms underlying the pathogenesis of CRC is essential for the advancement of effective prevention and therapeutic strategies.

Regulated cell death, often termed programmed cell death (PCD), constitutes a vital biological process occurring under physiological conditions. This process is crucial for the maintenance of tissue homeostasis and plays a complex role in various developmental processes. PCD includes several mechanisms, such as apoptosis, ferroptosis, autophagy, and necroptosis, each of which contributes uniquely to cellular regulation and organismal health.^7^ The regulation of various cell death pathways in CRC treatment holds potential for developing innovative strategies to enhance chemotherapy efficacy and address drug resistance.^8^^,^^9^ Numerous studies have demonstrated that specific small-molecule compounds can effectively target these pathways, thereby increasing the sensitivity of CRC cells to chemotherapeutic agents.^10^, ^11^, ^12^ In summary, a comprehensive understanding of the distinct forms of PCD and their implications in CRC is essential for the advancement of novel therapeutic approaches.

Nrf2 serves as the principal protector against oxidative damage through its interaction with antioxidant response elements (ARE) sequences.^13^ Beyond its role in cellular antioxidant defense, Nrf2 is integral to anti-inflammatory responses,^14^ cancer chemoresistance,^15^^,^^16^ and mitochondrial function.^17^^,^^18^ Elevated expression levels of Nrf2 have been observed in CRC tissues and certain CRC cell lines, where it is pivotal in sustaining redox homeostasis.^19^ The transcription factor Nrf2 regulates numerous genes crucial for antioxidant responses, essential in combating oxidative stress linked to cancer progression.^20^ In CRC progression, Nrf2 pathway activation plays a dual role: it protects normal cells from oxidative damage, offering a chemopreventive effect, but also aids cancer growth and therapy resistance by boosting antioxidant defenses in tumor cells, helping them survive in high-ROS conditions.^19^^,^^21^^,^^22^ Moreover, the interaction between Nrf2 and other regulatory proteins, including Kelch-like ECH-associated protein 1 (Keap1), plays a pivotal role in modulating its activity and stability, thereby influencing the overall redox balance in CRC cells.^19^^,^^23^ Numerous studies have indicated that excessive ROS production, from internal or external sources, can induce various forms of cell death in CRC cells.^24^, ^25^, ^26^, ^27^ Targeting Nrf2 activity to modulate this cell death may be significant for CRC treatment.

This work uniquely focuses on ROS as a link between Nrf2-mediated redox regulation and three key cell death pathways (apoptosis, ferroptosis, and autophagy) in CRC cells, unlike previous reviews. It presents a context-dependent framework for Nrf2’s dual role in protecting normal colonocytes from apoptosis during inflammation and promoting CRC chemoresistance by suppressing ferroptosis. Additionally, it critically examines combination therapy and offers underexplored actionable strategies. In this review, we will discuss Nrf2’s role in different cell death types related to oxidative stress. Understanding these complex regulatory networks is crucial for creating effective therapies targeting Nrf2 and related pathways in CRC treatment.

## Nrf2 signaling pathway and its role in CRC

### Regulation of Nrf2 expression and activity

Nrf2, also named NRF2 or NFE2L2, is a member of Cap’n’Collar (CNC) basic-region leucine zipper (bZIP) transcription factors and serves a key role in regulating cellular antioxidant response. Nrf2 consists of six Nrf2-ECH homology (Neh) domains, contributing to regulating the activity and function of Nrf2. The Neh1 domain contains a CNC-bZIP region that heterodimerizes with small musculoaponeurotic fibrosarcoma (sMaf) proteins to bind to DNA.^28^ Neh2 is located in the N-terminal of Nrf2, which is a highly conserved domain and interacts with Keap1 through binding with the DGR and CTR domains of Keap1.^29^ This results in proteasomal degradation of Nrf2 mediated by Keap1. Neh4 and Neh5 domains bind to CREB binding protein to transcriptionally activate Nrf2 target genes.^30^ Neh3 also acts as a transcriptional activation domain through binding to CHD6 protein.^31^ Neh6, rich in serine residues, is targeted by β-TrCP for Nrf2 degradation through glycogen synthase kinase 3β (GSK3β)-mediated phosphorylation.^32^

**Figure 1:**
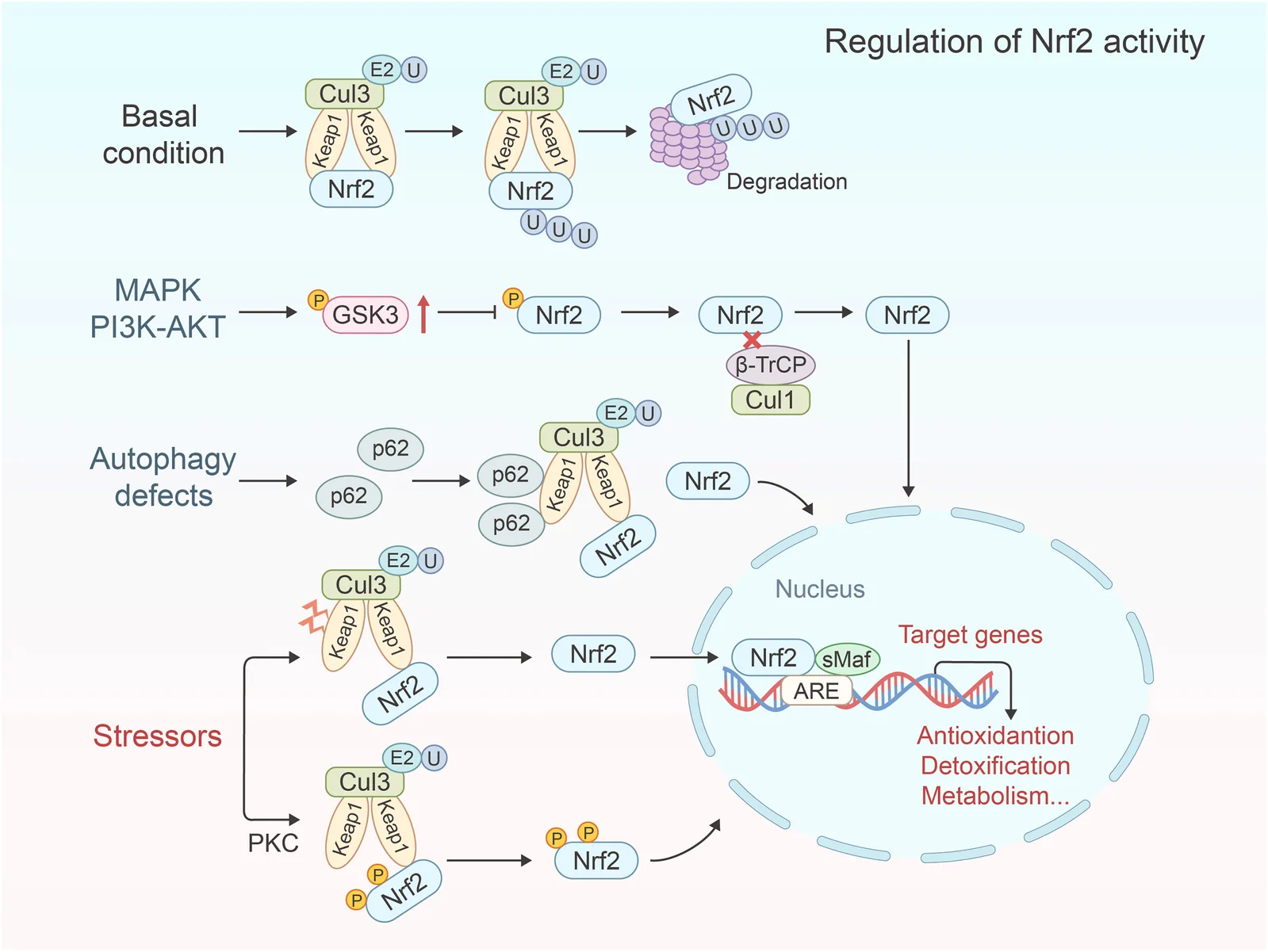
Regulation of Nrf2 activity. This figure emphasizes two main pathways for Nrf2 activation: Keap1-dependent (via oxidative stress-induced cysteine modification of Keap1) and Keap1-independent (through GSK3β/PI3K–AKT phosphorylation or p62-Keap1 competition), both regulating Nrf2’s degradation in the cytoplasm and movement to the nucleus. Under basal conditions, Nrf2 is ubiquitinated and degraded in the cytoplasm via the interaction between the Neh2 domain and the Keap1–Cullin3 complex. Exposure to electrophilic or oxidative stress leads to the modification of cysteine residues in Keap1 by oxidants or electrophiles, causing the release of Nrf2. Additionally, Nrf2 activity is regulated by GSK3β phosphorylation via the MAPK/PI3K–AKT pathway. The autophagy-related protein p62 can also activate Nrf2 by competing with it to bind to Keap1.

Based on the brief of Nrf2 domains, Neh2 and Neh6 are involved in regulating Nrf2 activity. As illustrated in Figure 1, under basal conditions, Nrf2 principally goes through ubiquitination and proteasomal degradation in the cytoplasm, which is mediated by the interaction between the Neh2 domain and Keap1–Cullin3 complex. This explains why basal Nrf2 levels are low.^33^ Upon exposure to electrophilic or oxidative stress, cells mitigate oxidative damage by activating the Nrf2–ARE pathway. Oxidants or electrophiles modify the cysteine residues in Keap1, leading to the release of Nrf2 from Keap1 (Fig. 1). Nrf2 then accumulates in the cytoplasm and translocates into the nucleus, where it forms heterodimers with sMaf proteins (MafF, MafG, MafK). These Nrf2–sMaf heterodimers bind to the ARE, activating target genes.^34^^,^^35^ Furthermore, numerous different types of Nrf2 activators have been described, and Nrf2 activation mainly depends on targeting cysteine residues in Keap1, and this can also be deemed to inhibit Keap1.^36^ Also, the mitogen-activated protein kinase (MAPK)/phosphoinositide 3-Kinase (PI3K)–protein kinase B (AKT) signaling pathway plays a crucial role in the regulation of Nrf2 activation by phosphorylating Ser9 of GSK3β. This phosphorylation event inhibits the GSK3β-mediated phosphorylation of Nrf2, thereby preventing the binding of β-TrCP to the Neh6 domain of Nrf2.^37^^,^^38^ This is also known as the Keap1-independent pathway to trigger Nrf2 (Fig. 1). Similarly, research demonstrates that stressors induce Nrf2 nuclear translocation and downstream gene activation relying on phosphorylated Ser40 in the Neh2 domain of Nrf2 by PKC.^39^ Furthermore, under conditions of autophagy dysfunction, the phosphorylation of the autophagy adaptor protein p62 can compete with Nrf2 for binding to Keap1. This competition results in the release of Nrf2 from Keap1, allowing its translocation into the nucleus.^40^

### Dual role of Nrf2 in CRC

Nrf2 has been broadly proven to be a major regulator of cytoprotection, metabolism, mitochondrial function, and cell proliferation. Among these effects, most studies focus on the antioxidant protection of Nrf2 throughout multiple tissues *in vivo* and *in vitro*. Under physiological state, ROS, including superoxide anion (O_2_^•–^), hydrogen peroxide (H_2_O_2_), hydroxyl radical (HO^•^), and singlet oxygen (^1^O_2_), mainly arise from endogenous sources such as mitochondria, endoplasmic reticulum, and peroxisomes.^41^^,^^42^ However, exposure to some exogenous stressors or pathological conditions, cells generate excessive ROS that leads to cellular damage and chronic diseases such as cancer, cardiovascular diseases, and neurodegenerative diseases.^43^, ^44^, ^45^, ^46^ Importantly, the Nrf2–ARE pathway is involved in antioxidant response and xenobiotic metabolism by activating antioxidant and phase II detoxication genes, including glutamate cysteine ligase catalytic (GCLC), glutamate cysteine ligase modifier (GCLM), and glutathione S-transferase (GST) to protect against oxidants and xenobiotic damage.^47^, ^48^, ^49^

Nrf2 functions dually as both a safeguard and an enhancer of tumorigenesis. On one hand, Nrf2 is recognized for its pivotal role as a master regulator of antioxidant genes, thereby mitigating oxidative stress and inflammation, both of which are critical factors in cancer development.^50^ This protective function is especially crucial in the context of CRC, where oxidative stress is prevalent due to the inflammatory milieu commonly associated with conditions such as inflammatory bowel disease.^51^, ^52^, ^53^ Conversely, Nrf2 activation may also result in detrimental effects in CRC. Numerous tumors, including CRC, demonstrate aberrant hyperactivation of Nrf2, a phenomenon associated with unfavorable prognostic outcomes and resistance to chemotherapy.^54^ Research indicates that Nrf2 boosts cytoprotective gene expression, potentially increasing chemoresistance in CRC cells.^55^^,^^56^ Thus, although Nrf2 protects against oxidative damage in intestinal epithelial cells, its dysregulation can promote CRC progression and treatment resistance. Table 1 summarizes the dual roles of Nrf2 in CRC, systematically comparing the contexts, mechanisms, and outcomes.

**Table 1:** Dual role of Nrf2 in CRC contexts.

CRC contexts	Mechanisms	Outcomes	Refs
Normal colonocytes/IBD	Nrf2 upregulates GCLC, GCLM and GPX3, reducing ROS and inflammation	Protects against oxidative damage; inhibits colitis-associated carcinogenesis	70, 52
Early-stage CRC	Nrf2 activates cytoprotective genes such as HO-1 and NQO1, suppressing apoptosis	Limits tumor initiation; maintains redox homeostasis	82, 148
Advanced CRC	Nrf2 overexpression caused by Keap1 mutation or p62 accumulation upregulates Bcl-2 and SLC7A11	Promotes chemoresistance; inhibits ferroptosis and apoptosis	54, 101
Normal tissue during therapy	Nrf2 activation reduces radiation-induced ferroptosis	Protects intestinal epithelium from radiation enteritis	110

Bcl-2, B-cell lymphoma-2; CRC, colorectal cancer; GCLC, glutamate cysteine ligase catalytic; GCLM, glutamate cysteine ligase modifier; GPX3, glutathione peroxidase 3; HO-1, heme oxygenase 1; IBD, inflammatory bowel disease; Keap1, Kelch-like ECH-associated protein 1; NQO1, NAD(P)H quinone oxidoreductase 1; Nrf2, Nuclear factor erythroid 2-related factor 2; ROS, reactive oxygen species; SLC7A11, solute carrier family 7 member 11.

### Apoptosis in CRC: ROS-mediated regulation and Nrf2’s modulatory role

#### *ROS-driven apoptotic signaling in CRC cells*

Apoptosis, a kind of programmed cell death, is regulated by a multitude of genes and signaling pathways. Apoptosis is split into extrinsic and intrinsic pathways.^57^^,^^58^ Both apoptosis pathways rely on caspases, essential proteins that are crucial for apoptosis.^58^ Meanwhile, nuclear factor kappa B (NF-κB), PI3K, and other pathways are also related to activating both extrinsic and intrinsic apoptotic pathways.^59^ Accumulation of misfolded proteins and disruption of calcium balance in the endoplasmic reticulum can cause endoplasmic reticulum stress, ultimately leading to apoptosis. The impact of oxidative stress on apoptosis in CRC is linked to the administration of inhibitors. The Wnt/β-catenin signaling pathway represents a crucial target for therapeutic intervention in colon cancer. Kim et al identified DGG-300273 as a novel inhibitor of the Wnt/β-catenin pathway, which induces ROS and promotes apoptosis through an ROS–BIM–caspase cascade in CRC cells.^60^ Natural compounds, exemplified by ursolic acid, synergistically induce ROS-dependent apoptosis in CRC cells by concurrently inhibiting multiple signaling pathways, including signal transducer and activator of transcription 3 (STAT3), extracellular signal-regulated kinase (ERK), and NF-κB. This multifaceted approach effectively addresses the challenge of single-target drug resistance.^61^

Various natural compounds, chemical elements, and biological extracts have been shown to influence apoptosis in CRC cells through mechanisms involving oxidative stress. Chlorogenic acid, an ester formed from caffeic acid and quinic acid, is one such compound. Research by Vélez-Vargas et al has demonstrated that chlorogenic acid can stimulate the production of mitochondrial ROS and induce apoptotic cell death via the Wnt/β-catenin signaling pathway in SW480 and HT29 cells.^62^ Phloretin is recognized as the principal dihydrochalcone polyphenol. Kapoor et al have shown that phloretin induces depolarization of the mitochondrial membrane potential (MMP) and disrupts the cell cycle by generating elevated levels of ROS, thereby facilitating apoptosis in CRC cells. Furthermore, treatment with phloretin altered the expression of apoptosis-related proteins in SW480 and HCT116 cells, specifically resulting in an up-regulation of the pro-apoptotic protein Bax and a down-regulation of the anti-apoptotic protein B-cell lymphoma-2 (Bcl-2).^63^ Plumbagin, a bioactive naphthoquinone derived from the Plumbaginaceae family, has been shown by Yadav et al to inhibit the Wnt signaling pathway through the up-regulation of miR-22-3p expression. Furthermore, both plumbagin and miR-22-3p were found to increase the protein levels of cleaved-PARP and decrease the protein levels of B-cell lymphoma-extra-large (Bcl-xL) in HCT116 and SW620 cells. This modulation of protein expression subsequently mediated apoptotic cell death via increased ROS production and decreased MMP.^64^ NO-ASA, a compound combining traditional aspirin with a nitric oxide-releasing component, shows potential against colon cancer. Gao et al found that NO-ASA inhibited intestinal cancer in APC^Min/+^ mice by triggering oxidative stress-induced apoptosis. *In vitro* studies revealed that NO-ASA activated caspase 9 and caspase 3, interrupting adherens junctions in SW480 cells.^65^ Cinnamomum cassia is a member of the Lauraceae family. According to the research conducted by Park et al, aqueous extracts derived from *Cinnamomum cassia* (referred to as TC-HW) have been shown to induce apoptosis in CRC cells. This apoptotic effect is mediated through the activation of ROS-dependent NF-κB signaling and activating transcription factor 3 (ATF3).^66^ Furthermore, certain drug combination therapies have demonstrated similar effects. Sokolov et al have reported that the concurrent administration of melatonin and andrographolide results in increased ROS levels, decreased MMP, and reduced ATP levels in colospheroids derived from HT29 cells. Additionally, this co-treatment suppresses the colospheroid phenotype and induces apoptosis by inhibiting the Wnt/β-catenin signaling pathway.^67^

Moreover, increasing the regulation of certain factors or enzymes can induce apoptotic cell death in CRC cells by elevating oxidative stress. Multitarget tyrosine kinase inhibitors are essential for treating metastatic CRC, with activin A receptor-like type 1 (ACVRL1) being a key gene in multitarget tyrosine kinase inhibitor resistance. Lu et al found that ACVRL1 stabilized glutathione peroxidase 2 (GPX2) via USP15-mediated deubiquitination, reducing ROS and apoptosis in CRC cells, thereby promoting multitarget tyrosine kinase inhibitor resistance. The findings from Lu et al indicate that targeting ACVRL1 may represent a promising strategy to mitigate this resistance.^68^ Peroxiredoxin 2 (Prdx2), an antioxidant enzyme, exhibits aberrantly elevated levels in CRC cells and tissues. In a study conducted by Lu et al, it was demonstrated that silencing Prdx2 enhanced the generation of ROS, thereby promoting apoptosis in CRC cells. This apoptotic effect is mediated through the down-regulation of Wnt/β-catenin signaling and the concomitant increase in ROS accumulation.^69^ Glutathione peroxidase 3 (GPX3), an extracellular member of the GPx family, functions as a tumor suppressor in the context of inflammatory colon carcinogenesis. Barrett et al demonstrated that *in vivo* knockdown of *GPX3* facilitated the development of colitis-associated carcinoma, correlating with elevated levels of ROS.^70^ Liu et al discovered that proline oxidase (POX), a flavoenzyme in the inner mitochondrial membrane, increased proline-dependent ROS to inhibit cyclooxygenase 2 (COX-2)/prostaglandin E2 (PGE2) signaling and trigger apoptosis in CRC cells.^71^

### Nrf2 as a context-dependent regulator of ROS-mediated apoptosis

While ROS mechanisms can trigger apoptosis in CRC cells by damaging mitochondria, increasing endoplasmic reticulum stress, or disrupting oncogenic signaling, CRC cells have developed strategies to limit ROS and prevent apoptosis. The transcription factor Nrf2 plays a key role in this defense by regulating antioxidant and protective genes, influencing whether cells survive or become more sensitive to apoptosis. Understanding the interaction between ROS-induced apoptosis and Nrf2 adaptation is crucial for targeting these processes in cancer therapy.

Tumor necrosis factor receptor-associated protein 1 (TRAP1), a member of mitochondrial heat shock protein, is crucial in mitigating oxidative stress and apoptosis by modulating ROS levels. The inhibition of TRAP1 in CRC cells results in the generation of ROS, endoplasmic reticulum stress, decreased cell viability, and ultimately, cell death. The suppression of Nrf2 intensifies endoplasmic reticulum stress and cell apoptosis triggered by the TRAP1 inhibitor G-TPP via the overproduction of ROS.^72^ Co-administering aspirin and zinc enhances Nrf2 activation, boosting antioxidant defenses in tumor cells and protecting against DMH-DSS-induced colitis-associated CRC by targeting inflammation, oxidative stress, cell proliferation, and apoptosis.^73^ However, Nrf2 activation can hinder tumor suppressor p53 and decrease CRC cell death by reducing DNA damage. ZnCl_2_ supplementation can inhibit Nrf2 activation, suggesting that inhibiting Nrf2 could be a promising anti-cancer strategy, with ZnCl_2_ beneficially suppressing the Nrf2 pathway in combination therapies.^74^ The lncRNA MIR4435-2HG plays a pivotal role in the development of cisplatin resistance in HCT116 cells, with its mechanism of action involving the Nrf2/HO-1 pathway. Knockdown of *MIR4435-2HG* in cisplatin-resistant HCT116 cells leads to enhanced sensitivity to cisplatin, suppression of cell proliferation, and induction of apoptosis.^75^ Sestrin2 (SESN2), identified as a novel CRC suppressor, exhibits dual functions in tumor suppression. Hemin triggers ROS in CRC cells, activating Nrf2 to increase SESN2 expression based on dose. This Nrf2-driven SESN2 rise acts as feedback to reduce oxidative stress from hemin. Typically, SESN2 overexpression limits CRC cell growth by lowering oxidative stress, but with hemin, Nrf2-induced SESN2 up-regulation hinders hemin-induced apoptosis, thus promoting CRC progression in high-iron environments.^76^

**Figure 2:**
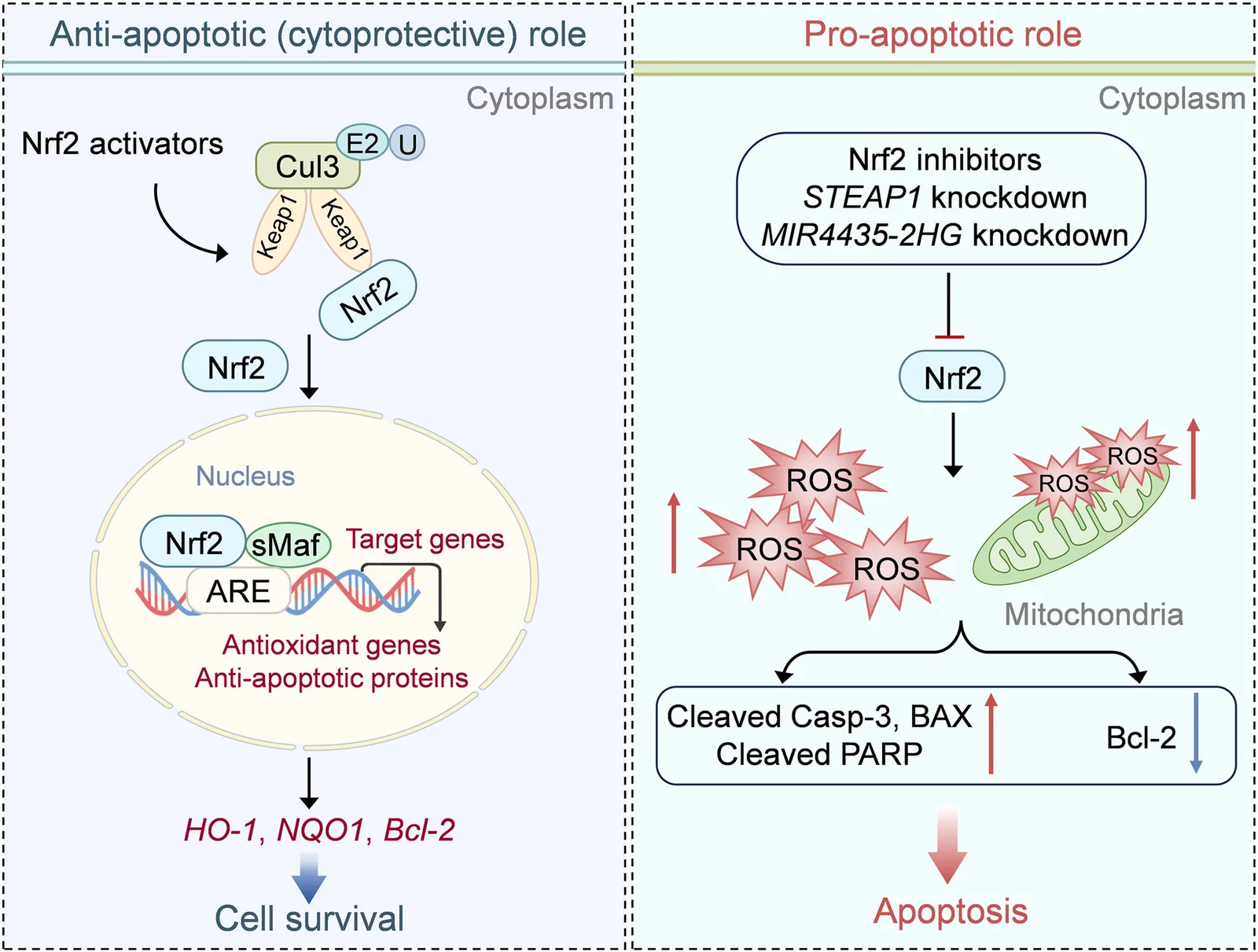
Nrf2’s dual role in regulating apoptosis in colorectal cancer (CRC) cells. This figure illustrates Nrf2’s context-dependent effects on CRC apoptosis: Anti-apoptotic role (left panel): In colitis-associated CRC or chemoresistant cells, Nrf2 activation (via aspirin-zinc co-treatment, *STEAP1* overexpression, or acetazolamide) enhances antioxidant genes and anti-apoptotic proteins, reduces ROS and DNA damage, leading to decreased apoptosis and increased tumor survival. Pro-apoptotic role (right panel): Nrf2 inhibition (*MIR4435-2HG* knockdown, *STEAP1* knockdown, marine peroxy sesquiterpenoids, or metformin) increases ROS, lowers Bcl-2, and activates pro-apoptotic pathways, promoting apoptosis in CRC cells.

The pathogenesis of a significant proportion of human cancers is attributed to the overexpression of six-transmembrane epithelial antigen of the prostate 1 (STEAP1). Elevated levels of STEAP1 transcripts in CRC cells have been found to diminish ROS production and impede apoptosis by activating the Nrf2 pathway. Conversely, silencing of *STEAP1* in CRC cells triggers apoptosis by inducing an excessive accumulation of ROS due to the suppression of Nrf2-mediated antioxidant mechanisms.^77^ Marine peroxy sesquiterpenoids exhibited anti-tumor effects in HCT116 cells by inhibiting the Nrf2–ARE pathway. These compounds down-regulated the expression of Nrf2, pNrf2, and HO-1 via the PI3K–AKT pathway, ultimately inducing apoptosis by suppressing the anti-apoptotic protein Bcl-xL.^78^ Manuka honey exhibited significant antioxidant properties and augmented 5-fluorouracil-induced apoptosis in HCT116 and LoVo cells by suppressing the expression of Nrf2 and NF-κB family proteins, as well as antioxidant enzyme activity. This resulted in elevated levels of ROS and pro-apoptotic proteins.^79^ Metformin, also known as 1,1-dimethylbiguanide hydrochloride, is a biguanide derivative that has been shown to impede the progression of CRC by suppressing the transcriptional activation of Nrf2 and NF-κB in HT29 cells in a manner that is contingent upon both dosage and duration, ultimately resulting in apoptosis in HT29 cells.^80^ Certain carbonic anhydrase (CA) isozymes have been implicated in carcinogenesis, with acetazolamide identified as an inhibitor of CA. In APC-mutant mice, acetazolamide has been shown to inhibit the development of intestinal polyps and enhance the apoptosis ratio in the intestinal polyps. This effect is mediated by acetazolamide’s ability to enhance Nrf2 transcriptional activity, subsequently suppressing the expression of inflammation-related genes such as IL-6^81^ (Fig. 2).

Furthermore, Nrf2 likely plays a significant role in the development of colitis-associated cancer in its early stages. Inflammatory myeloid cells in the colon of individuals with inflammatory bowel disease induce a transient rise in ROS in NCM460 colonocytes, triggering Nrf2 activation. This activation results in heightened proteasome activity and protection against apoptosis.^82^ Liu et al discovered that curcumin, a phytochemical derived from turmeric roots, activated miR-34a and miR-34b/c as key effectors of the ROS/Nrf2 pathway. This activation led to apoptosis in CRC cells and inhibited metastasis in a manner dependent on ROS/Nrf2 signaling^53^ (Fig. 2). Genetic polymorphisms have been identified as significant contributors to cell death in CRC cells. Chen et al demonstrated a correlation between genetic polymorphisms in Nrf2 and apoptosis in CRC cells, highlighting their crucial involvement in the prognostic outcomes of rectal cancer patients undergoing postoperative chemoradiotherapy.^83^ Jeong et al unveiled that AF8c, a derivative of lapatinib, induced apoptosis through a mechanism involving heightened mitochondrial ROS production and consequent activation of DR5-dependent Nrf2. Furthermore, AF8c was observed to up-regulate Nrf2 expression in a manner dependent on p53, ultimately resulting in apoptosis.^84^ Table 2 and Figure 2 provide summaries of the primary references discussed in this section.

**Table 2:** Effects of Nrf2 activity on the apoptotic properties of CRC cells.

Cell/Animal models	Nrf2 manipulation	Apoptosis-related indicators	Effects	Refs
SW48, DLD-1, RKO	↓, Pharmacologic (ML385)	Caspase-3, -7, CHOP, Cleaved PARP	Reduce drug resistance	72
Male BALB/c mice	↑, Pharmacologic (aspirin + ZnSO_4_)	IL-6, STAT3	Inhibit proliferation	73
HCT116, HCT116-p53^−/−^	↓, Genetic (KO: Nrf2)	p53	Inhibit proliferation; Reduce drug resistance	74
HCT116	↓, Genetic (MIR4435-2HG siRNA)	Caspase-3	Reduce drug resistance; Inhibit proliferation	75
DLD-1, HCT116, SW480	↓, Genetic (STEAP1 siRNA)	Caspase-9	Inhibit proliferation	77
HCT116	↓, Pharmacologic (Marine Peroxy Sesquiterpenoids)	Bcl-xL	Inhibit proliferation	78
HCT116, LoVo	↓, Pharmacologic (MH+5-FU)	p53, Bax, Cyto c, FasL, Caspase-3, -8, -9 and Cleaved PARP	Reduce drug resistance; Inhibit proliferation; Inhibit invasion	79
HT29	↓, Pharmacologic (Metformin)	APAF-1, Cleaved PARP-1, Caspase-3	Inhibit proliferation	80
SW48, HCT15	↑, Pharmacologic (Acetazolamide)	Bcl-2	Inhibit proliferation	81
HCT116, RKO, SW48, CCD-18Co, SW620-Luc2	↑, Pharmacologic (Curcumin)	Cleaved PARP, Cleaved caspase-3, Bcl-2, Bax	Inhibit invasion	53
HT29, HCT116	↑, Pharmacologic (AF8c)	Cleaved PARP, Caspase-3, -8, -9	Inhibit proliferation	84

APAF-1, apoptosis-activating factor-1; Bax, Bcl-2 associated X protein; Bcl-xL, B-cell lymphoma-extra-large; c-PARP, cleaved-poly (ADP-ribose) polymerase; Cyto c, cytochrome c; CRC, colorectal cancer; FasL, fatty acid synthetase ligand; IL-6, interleukin-6; MH, manuka honey; Nrf2, Nuclear factor erythroid 2-related factor 2; STAT3, signal transducer and activator of transcription 3; STEAP1, six-transmembrane epithelial antigen of the prostate 1; “↑” or “↓”mean Nrf2 activation and inhibition, respectively.

Although ROS effectively induces apoptosis in CRC cells through mechanisms such as mitochondrial ROS-induced MMP depolarization,^63^ BIM–caspase cascade activation,^60^ or Wnt/β-catenin pathway inhibition,^69^ CRC cells, particularly in advanced stages or under chemotherapy, develop adaptive defenses to limit this apoptosis. Nrf2 is a key player in adaptation by activating antioxidant genes and influencing apoptosis-related proteins, linking redox balance to cell death. Its role varies with context: in normal colonocytes or early CRC, it reduces ROS to prevent apoptosis and tumor initiation; in advanced CRC, constant Nrf2 activation increases Bcl-xL, reducing caspase activity and promoting chemoresistance. Understanding the Nrf2–ROS–apoptosis interaction across CRC stages and genetics is crucial for developing therapies that enhance ROS-mediated apoptosis or block Nrf2’s protective effects.

### Ferroptosis in CRC: ROS, lipid metabolism, and Nrf2-driven resistance

#### *ROS and lipid peroxidation:* C*ore triggers of ferroptosis in CRC*

Ferroptosis, a form of PCD dependent on iron and ROS, is characterized by suppressing the accumulation of glutathione peroxidase 4 (GPX4) and promoting the production of lipid peroxides within cells, ultimately resulting in the demise of drug-resistant tumor cells. GPX4 plays a crucial role in regulating ferroptosis and possesses antioxidant properties. The process of ferroptosis involves the inactivation of GPX4, a depletion of reducing agents within cells, and an imbalance in intracellular redox homeostasis. The oxidative metabolism of mitochondria contributes to the accumulation of ROS, thereby facilitating the death of CRC cells.^85^ Furthermore, the utilization of ferroptosis as a treatment modality has emerged as a promising strategy to combat drug resistance in tumor cells. The administration of ferroptosis inducers either as a standalone treatment or in conjunction with traditional chemotherapeutic agents has shown efficacy in inducing ferroptosis in drug-resistant tumor cells, thereby presenting a novel therapeutic approach for addressing drug resistance in CRC.^86^^,^^87^

In CRC cells, oxidative stress modulation affects ferroptosis. Key genes like *GPX4* and the cystine transporter *SLC7A11*, which are crucial for lipid metabolism and redox balance, significantly influence ferroptosis and CRC progression.^88^^,^^89^ Gao et al have demonstrated that the copper carrier elesclomol effectively induces copper retention through the degradation of the copper transporter ATP7A. This excessive accumulation of copper disrupts mitochondrial respiration, resulting in the generation of ROS in CRC cells. The elevated ROS levels subsequently promote the degradation of SLC7A11, ultimately leading to ferroptosis in CRC cells.^90^ Moreover, the metal-based complex (Cu-1) has been shown to enhance anti-tumor immunity in CRC cells. Cu-1 disrupts redox homeostasis and induces ferroptosis-dependent cell death both *in vivo* and *in vitro*.^26^ Nikolova et al have shown that treating Colon26 cells with both camptothecin analog SN38 and electroporation raises ROS levels and disrupts redox homeostasis, lowering intracellular glutathione (GSH) and increasing lipid peroxidation, which induces ferroptosis.^91^

Ferroptosis in CRC cells can be triggered by boosting regulatory factors or enzymes that heighten oxidative stress. Lei et al clarified that the tumor suppressor gene APC membrane recruitment 1 (AMER1) induced ferroptosis by promoting the ubiquitination and degradation of SLC7A11 and FTL, altering intracellular cystine and Fe^2+^ levels. *AMER1* deficiency, however, inhibited ferroptosis under elevated oxidative stress levels, thereby facilitating CRC metastasis.^92^ These findings suggest a potential treatment for metastatic CRC patients with AMER1 mutations. Additionally, Sinha et al discovered that NCX4040, a non-steroidal nitric oxide donor, triggered oxidative stress, dose-dependent lipid peroxidation, and ferroptosis-related gene expression, leading to ferroptosis in CRC cells.^93^ Singhal et al revealed that hypoxia-inducible factor 2-alpha (HIF-2α) activation in CRC cells and specific mouse models up-regulated lipid and iron regulatory genes, making cells more prone to ferroptosis. Furthermore, HIF-2α elevated ROS levels, causing oxidative cell death through irreversible cysteine oxidation.^94^ Further, excessive ROS production under certain enzyme treatment can cause PANoptosis, composed of apoptosis, necroptosis, pyroptosis, and ferroptosis in CRC cells. Lin et al revealed that a lack of cysteine desulfurase (NFS1) combined with oxaliplatin treatment triggered PANoptosis by raising intracellular ROS levels, making CRC cells more sensitive to the drug. Oxidative stress is crucial for this process. Additionally, oxaliplatin-induced oxidative stress increased NFS1 serine phosphorylation, which inhibited PANoptosis in a phosphorylation-dependent manner.^95^ Exploring the interplay between oxidative stress and ferroptosis may lead to new treatment strategies for CRC by focusing on related genes. Lorenzato et al found that combining vitamin C with cetuximab prevented resistance to EGFR-targeted therapy in CRC. Vitamin C also disrupted iron balance and raised ROS levels, inducing ferroptosis.^96^ The aforementioned findings indicate that ROS-inducing drugs, which modulate ferroptosis, could enhance the effectiveness of chemotherapy in treating CRC.

### Nrf2-mediated suppression of ROS-lipid peroxidation to antagonize ferroptosis

Targeting Nrf2, which plays a role in anti-oxidative stress, could be an effective approach to regulate ferroptosis in tumor cells. By influencing its downstream antioxidant genes, Nrf2 can reduce ferroptosis, aiding tumor cell survival while protecting normal cells. Research has demonstrated that Nrf2 activation in tumor cells promotes cancer progression, metastasis, and resistance to treatments. Thus, drugs that induce ferroptosis by targeting Nrf2 are being developed for cancer therapy.^97^ Sun et al found that brusatol, an Nrf2 inhibitor, induced ferroptosis in hepatocellular carcinoma cells, increasing their sensitivity to sorafenib by suppressing metallothionein-1G (MT-1G), a lipid peroxidation regulator.^98^ The suppression of Nrf2 by trigonelline has been shown to enhance the susceptibility of head and neck cancer cells to ferroptosis induced by RSL3 and artesunate.^99^^,^^100^

The enzyme uridine-cytidine kinase-like-1 (UCKL1), a key player in the pyrimidine salvage pathway, has been found to be significantly up-regulated in various types of cancer. Recent research by Wu et al has revealed that UCKL1 plays a crucial role in stabilizing the transcription factor Nrf2, leading to increased expression of SLC7A11 in CRC cells. SLC7A11 is essential for the production of GSH, which is responsible for detoxifying lipid peroxides and preventing ferroptosis in conjunction with GPX4. Therefore, targeting UCKL1 is essential for inducing ferroptosis through the UCKL1–Nrf2–SLC7A11 axis in CRC cells, offering a promising approach for the treatment of CRC.^101^ Sodium butyrate (NaB) down-regulates the expression of SLC7A11 via the FFAR2–AKT–Nrf2 pathway, thereby inhibiting the synthesis of GPX4 and inducing lipid ROS production, which promotes ferroptosis in CRC cells. *In vivo*, NaB treatment mitigated CRC progression in a model of colitis-associated colorectal tumorigenesis by reducing the expression of Nrf2 and SLC7A11. These findings suggest that NaB modulates the development of CRC through a ferroptosis-dependent mechanism.^102^ KRAS mutations are present in about 40% of CRC cases and lead to resistance against anti-EGFR therapies like cetuximab, limiting their effectiveness in these patients. The study by Müller et al highlighted that Nrf2 activity affected the sensitivity of KRAS-mutant CRC cells to ferroptosis. Oncogenic KRAS increases ferroptosis suppressor protein 1 (FSP1) through the MAPK–Nrf2 pathway, protecting these cells from ferroptosis. Therefore, targeting Nrf2 to induce ferroptosis could be a promising treatment strategy for KRAS-mutant CRC.^103^ Preclinically, however, cetuximab has been shown to suppress the p38 MAPK–Nrf2–HO-1 pathway in KRAS-mutant CRC cells, reducing GPX4 expression and sensitizing cells to ferroptosis.^104^ This suggests potential for cetuximab in combination therapies, but further validation in KRAS-mutant CRC models and clinical trials is needed.

**Figure 3:**
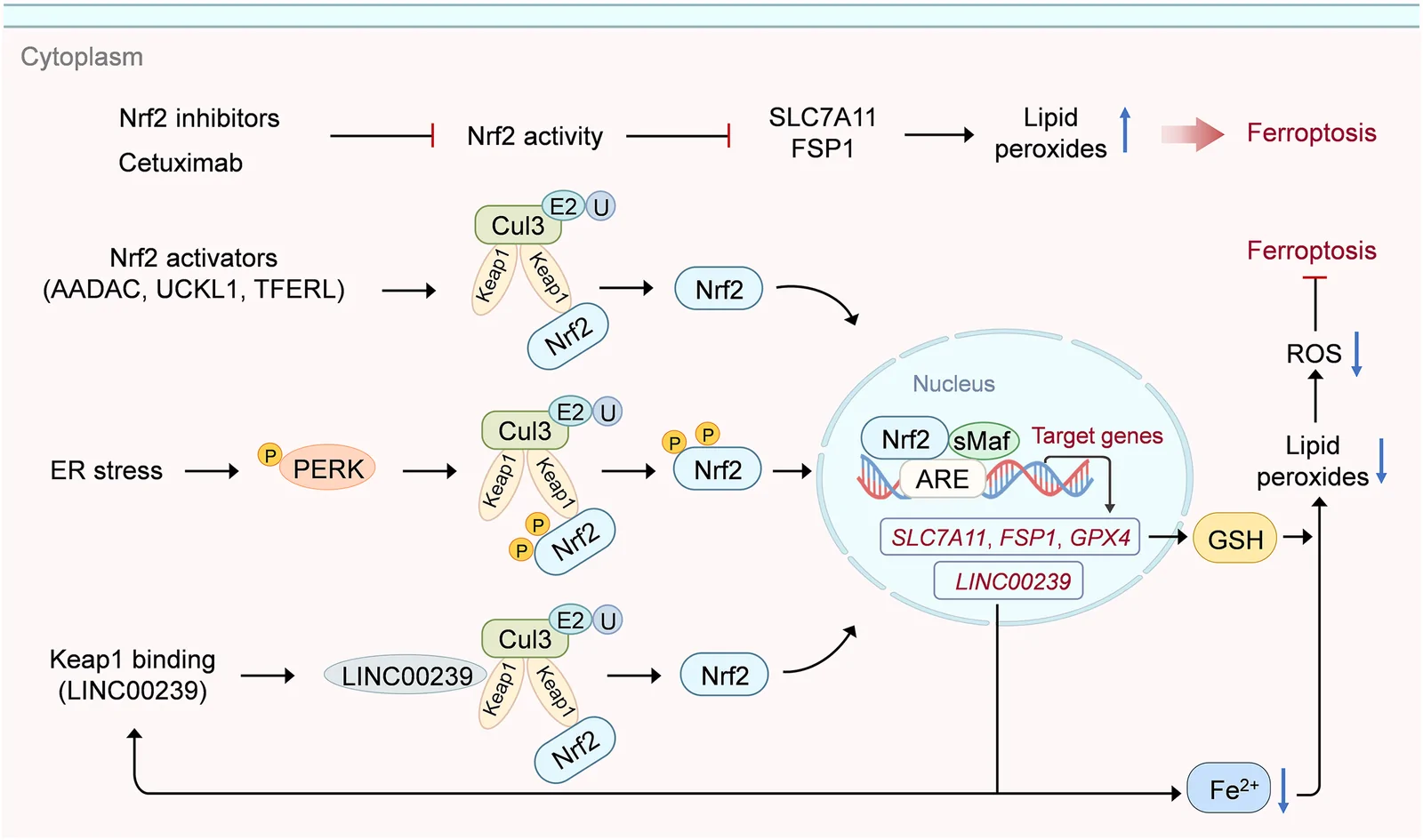
Nrf2-mediated ferroptosis resistance in colorectal cancer cells and sensitization strategies. The activation of Nrf2 during ferroptosis in colorectal cancer cells primarily occurs through a Keap1-dependent pathway. This activation results in the up-regulation of downstream target genes, including *SLC7A11*, *FSP1*, *GPX4*, and *LINC00239*. Additionally, LINC00239 interacts with Keap1, thereby enhancing Nrf2 levels and establishing a positive feedback regulatory mechanism. Ultimately, this process contributes to the reduction of intracellular lipid peroxides and the inhibition of ferroptosis. Upper panel highlights strategies to restore ferroptosis: Nrf2 inhibitors reduce SLC7A11/GPX4, while cetuximab disrupts the KRAS–MAPK–Nrf2–FSP1 axis in KRAS-mutant colorectal cancer cells.

Moreover, research has demonstrated that Nrf2-mediated ferroptosis in CRC cells significantly contributes to the process of liver colonization in CRC. Arylacetamide deacetylase (AADAC) up-regulated SLC7A11 through the activation of Nrf2, resulting in the inhibition of lipid peroxidation. This mechanism conferred protection to CRC cells against ferroptosis, thereby facilitating their colonization in the liver.^105^ Elevated expression of the long noncoding RNA LINC00239 is positively associated with poor prognosis in CRC patients, primarily through the inhibition of ferroptosis. LINC00239 impedes the ubiquitination of Nrf2 and enhances its stability by interacting with Keap1, thereby inhibiting ferroptosis in CRC cells.^106^ The research conducted by Liu et al demonstrated that oxaliplatin induced ferroptosis and oxidative stress in HT29 CRC cells by inhibiting Nrf2 and its downstream antioxidant genes. Consequently, the inhibition of Nrf2 represents a critical regulatory mechanism that facilitates the induction of ferroptosis.^107^ Yuan et al demonstrated that elevated iron levels may elevate the risk of CRC by modulating the Warburg effect and ferroptosis. Iron exposure was shown to induce ROS and activate Nrf2, which in turn up-regulated the expression of glycolysis-related genes and GSH-related proteins, specifically SLC7A11 and GPX4. This regulatory pathway enhances the Warburg effect and confers protection to CRC cells against ferroptosis^108^ (Fig. 3).

Traditional Chinese medicine and plant extracts contribute significantly to CRC treatment by targeting Nrf2 to regulate ferroptosis. Ginsenoside Rh3 (GRh3), derived from Chinese herbs, inhibits Nrf2 activation, which suppresses SLC7A11 and induces ferroptosis in CRC cells.^109^ In the context of radiation-induced intestinal injury, the activation of the Nrf2 signaling pathway and the inhibition of ferroptosis demonstrate protective effects on normal intestinal epithelial cells. Wu et al uncovered that the total flavonoids of *Engelhardia roxburghiana* Wall. leaves (TFERL) mitigated radiation-induced intestinal injury by up-regulating the expression of Nrf2 and its downstream proteins. Furthermore, TFERL enhanced the expression of GPX4, resulting in decreased lipid peroxidation and inhibition of ionizing radiation-induced ferroptosis.^110^ Based on the findings of the study conducted by Wei et al, tagitinin C, a natural compound derived from *Tithonia diversifolia*, exhibits potential as a therapeutic agent for CRC. The administration of tagitinin C was observed to induce lipid peroxidation and oxidative stress by enhancing the production of MDA and ROS in HCT116 cells. Additionally, tagitinin C was found to trigger endoplasmic reticulum stress, which was mediated by the activation of the Nrf2–HO-1 pathway. Consequently, tagitinin C induced ferroptosis in CRC cells through endoplasmic reticulum stress-mediated activation of the PERK–Nrf2–HO-1 signaling pathway.^111^ Table 3 and Figure 3 provide summaries of the primary references discussed in this section.

**Table 3:** Effects of Nrf2 activity on the ferroptosis properties of CRC cells.

Cell/Animal models	Nrf2 manipulation	Ferroptosis-related indicators	Effects	Refs
HCT116, RKO	↓, Genetic (UCKL1 shRNA)	GSH, GPX4, FSP1	Inhibit proliferation	101
HT29, HCT116	↓, Pharmacologic (NaB)	GPX4, SLC7A11	Inhibit proliferation	102
HCT116, DLD-1	↓, Pharmacologic (Cetuximab)	MDA, Lipid ROS, Intracellular iron	Inhibit proliferation	104
HCT116, SW480	↑, Genetic (AADAC-OE)	GSH/GSSG, MDA, SLC7A11	Promote proliferation; Promote invasion	105
HCT116, SW620	↑, Genetic (LINC00239-OE)	GSH/GSSG, GPX4, Lipid ROS	Promote proliferation	106
HT29	↓, Pharmacologic (Oxaliplatin)	GPX4, FTH1, MDA, GSH	Inhibit proliferation	107
HCT116, HT29	↑, Pharmacologic (FAC)	SLC7A11, GPX4	Promote proliferation	108
HT29, HCT116	↓, Pharmacologic (GRh3)	SLC7A11, GSH, MDA	Inhibit proliferation	109
C57BL/6 mice	↑, Pharmacologic (TFERL)	GPX4	Promote proliferation	110
HCT116	↑, Pharmacologic (Tagitinin C)	MDA, LIP, Lipid peroxidation	Inhibit proliferation; Inhibit invasion	111

AADAC, arylacetamide deacetylase; CRC, colorectal cancer; FAC, ferric ammonium citrate; FSP1, ferroptosis suppressor protein 1; FTH1, ferritin heavy chain 1; GPX4, glutathione peroxidase 4; GRh3, Ginsenoside Rh3; GSH, glutathione; GSSG, oxidized glutathione; LIP, labile iron pool; MDA, malondialdehyde; NaB, sodium butyrate; Nrf2, Nuclear factor erythroid 2-related factor 2; OE, over expression; ROS, reactive oxygen species; SLC7A11, solute carrier family 7 member 11; TFERL, The total flavonoid of Engelhardia roxburghiana wall. leaves; UCKL1, uridine-cytidine kinase like-1; “↑” or “↓”mean Nrf2 activation and inhibition, respectively.

Despite progress in understanding Nrf2’s role in preventing ferroptosis through the SLC7A11–GSH–GPX4 pathway, significant gaps persist. Most studies use engineered cell lines or xenografts, with limited data from CRC patient-derived organoids that better reflect tumor diversity, especially in KRAS-mutant CRC, where Nrf2-driven FSP1 up-regulation leads to ferroptosis resistance. Whether Nrf2 directly affects ACSL4 or influences lipid peroxidation through non-GSH pathways like TRX1 needs further study. Additionally, there is a lack of *in vivo* data on Nrf2’s role in patient-derived xenografts, as current research mainly uses cell lines. The potential risk of Nrf2 inhibitors worsening radiation-induced intestinal ferroptosis is unclear. Addressing these issues requires targeted preclinical testing and redox biomarkers to aid clinical application.

### Autophagy in CRC: Nrf2’s dual control of cytoprotection and cell death

#### *ROS as a key inducer of autophagic flux in CRC*

Autophagy is a cellular process responsible for the intracellular degradation and the maintenance of cellular homeostasis. During this process, cytoplasmic components are transported to lysosomes, where they undergo degradation and subsequent recycling.^112^ Autophagy is vital for cellular homeostasis and stress adaptation, regulated by oxidative processes. Research increasingly shows that ROS stimulates autophagy, particularly in cancer development.^113^^,^^114^ Accumulating evidence shows that lysosomes are crucial in tumorigenesis. Anlotinib, a receptor tyrosine kinase inhibitor, boosts the expression of lysosome-related genes like ATP6V0E2 and TFEB, enhancing lysosomal function and autophagosome fusion in CRC cells. Inhibiting lysosomal function increases anlotinib-induced cell death and tumor suppression, likely due to elevated ROS levels. Thus, combining anlotinib with lysosomal inhibitors could be a promising new treatment for colon cancer.^115^ Chen et al found that combining the autophagy inhibitor chloroquine with SN-38 induces a ROS burst in CRC cells, with ROS and p53 mutually enhancing each other. Chloroquine enhances the sensitivity of CRC cells to SN-38/CPT-11 through lysosomal and mitochondrial apoptotic pathways via p53-ROS interaction.^116^ Autophagy enhancers can facilitate autophagy through the induction of ROS. Huang et al demonstrated that IR-58, a novel mitochondria-targeting near-infrared (NIR) autophagy enhancer, induced excessive ROS and autophagy via the TIM44–SOD2–ROS–mTOR pathway. Furthermore, IR-58 induced CRC cells’ death through the ROS–AKT–mTOR pathway by mediating excessive autophagy.^117^

Certain ingredients derived from animals, plants, and traditional Chinese medicine, along with various organic compounds and their derivatives, have been found to promote autophagy by inducing the production of excess ROS. Wang et al have demonstrated that β-elemene from curcuma elevates ROS levels, enhances AMPK phosphorylation, and inhibits mTOR phosphorylation in CRC cells, thereby inducing autophagy via the ROS/AMPK/mTOR pathway.^118^ Bromelain, a pineapple extract, has been found to induce elevated levels of ROS and superoxide, as well as the formation of autophagosomes and lysosomes. This process inhibited CRC cell proliferation by promoting programmed autophagic cell death.^119^ Aescin, a triterpene saponin, has been shown by Li et al to boost TIGAR expression, elevate ROS levels, and trigger autophagy. In HCT116 cells, knocking down TIGAR enhances aescin-induced ROS, autophagy, and its anti-cancer effects.^120^ Additionally, Silibinin acts as a “double-edged sword” for CRC cells. Raina et al demonstrated that it triggered ROS production and oxidative stress in SW480 cells, leading to endoplasmic reticulum stress-mediated autophagic death.^121^ It has been reported that certain compounds induce autophagy by disrupting mitochondrial balance and increasing intracellular ROS. Artesunate, an autophagy inducer, caused mitochondrial dysfunction and boosted mitochondrial ROS, thereby promoting autophagy and exerting an anti-CRC effect, according to Huang et al.^122^ Compound 6c, a novel naphthalimide derivative, inhibited isocitrate dehydrogenase 2 (IDH2), thereby inducing mitochondrial dysfunction and oxidative stress. This resulted in the accumulation of ROS in HCT116 and HT29 cells, ultimately leading to autophagic cell death in CRC cells in a ROS-dependent manner.^123^ In addition, Zhang et al found that dendrobium officinale polysaccharide (DOP) disrupted mitochondrial function by raising ROS levels and lowering MMP. This activated AMPK/mTOR autophagy signaling and inhibited CT26 cell proliferation.^124^

Moreover, studies indicate that high ROS levels in CRC cells can initiate cytoprotective autophagy, reducing the effectiveness of some anticancer drugs. Zhou et al have discovered that the anti-cancer effects of ciclopirox olamine (CPX) are linked to DJ-1 down-regulation, causing mitochondrial dysfunction and ROS buildup, which triggers protective autophagy in CRC cells. The study suggests that combining CPX with an autophagy inhibitor could boost its anti-CRC effectiveness.^125^ BCG/CWS is the cell wall skeleton of Bacillus Calmette-Guerin, which has effective anti-tumor immunotherapy effects. Yuk et al found that it enhanced CRC radiosensitivity by inducing autophagy through ROS generation when combined with ionizing radiation.^126^ BRG1, an ATPase in the SWI/SNF complex, crucially prevents inflammation-related CRC by regulating autophagosome formation. Liu et al found that BRG1 reduced colitis and CRC by managing autophagy-related oxidative stress in *Brg1* knockout mice.^127^

In addition, some studies have shown that innovative therapies can significantly increase ROS production in CRC cells, triggering autophagy and ultimately producing anti-tumor effects. Photodynamic therapy is a minimally invasive and effective cancer treatment. Meta-tetrahydroxyphenylchlorin and verteporfin are promising sensitizers in photodynamic therapy, showing significant phototoxicity by inducing autophagy in CRC cells via ROS up-regulation and activation of the ROS/JNK pathway.^128^ TRAIL/Apo2L resistance in CRC therapy is often due to low levels or mutations of TRAIL receptors. Shi et al showed that NanoTRAIL, which combines TRAIL/Apo2L with iron oxide nanoparticles, activated ROS-triggered JNK and induced autophagy-assisted DR5 up-regulation, significantly boosting the anti-tumor effects of TRAIL/Apo2L.^129^ Besides, Ba et al found that combining radiotherapy with hyperthermia in CRC cells boosted intracellular ROS, Beclin1, and LC3B levels, triggering autophagy. This indicates that hyperthermia promotes autophagic cell death via ROS and increases cell sensitivity to radiotherapy.^130^

### Nrf2–P62 feedback loop: Modulating ROS-dependent autophagy outcomes

**Figure 4:**
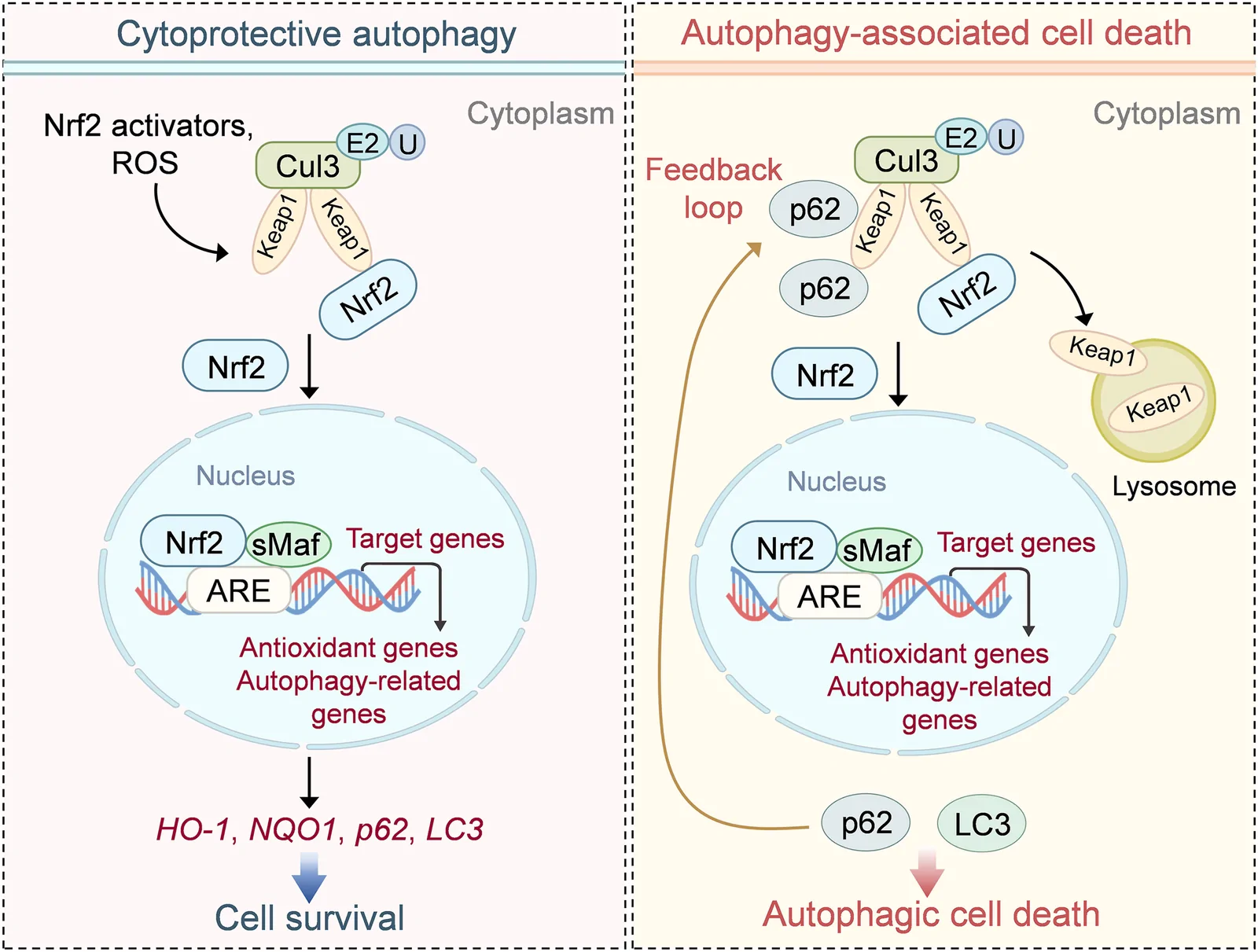
Nrf2 activation pathways and their regulatory mechanisms in the autophagy of colorectal cancer cells. Cytoprotective autophagy (left panel): The Keap1-dependent pathway constitutes the primary mechanism for Nrf2 activation involved in autophagy within colorectal cancer cells. This process encompasses Nrf2 activators and ROS. Consequently, the degradation of Nrf2 via the ubiquitin-proteasome pathway is diminished. Upon translocation into the nucleus, Nrf2 facilitates the transcription of downstream antioxidant genes, including *HO-1* and *NQO1*, thereby enhancing cellular survival mechanisms. Autophagy-associated cell death (right panel): Nrf2 up-regulates the expression of p62, which subsequently interacts with Keap1 to further activate Nrf2, establishing a positive feedback loop. Moreover, Nrf2 activation is associated with an up-regulation of the autophagy-related protein LC3, which leads to excessive autophagy and cell death.

Nrf2 regulates autophagy both directly and indirectly. It directly induces autophagy-related genes like Atg5, p62, LC3B, and ULK1.^131^^,^^132^ Indirectly, it activates selective autophagy by inducing Sestrin2, which inhibits the expression of mTORC1.^132^ Furthermore, in basal circumstances, Nrf2 undergoes ubiquitination facilitated by Keap1, followed by degradation via proteasomes. However, in the presence of oxidative stress, the interaction between Keap1 and Nrf2 is disrupted, thereby liberating Nrf2. Subsequently, Nrf2 is translocated to the nucleus, which triggers the expression of a multitude of Nrf2 target genes.^133^ Upon translocation of Nrf2 to the nucleus, the target gene p62 is activated. Moreover, p62 contains a Keap1 interaction region, which includes the Keap1 domain responsible for Nrf2 interactions. Phosphorylated p62 exhibits a higher affinity for Keap1 compared with the ETGE motif of Nrf2. This phosphorylated p62 physically interacts with Keap1, thereby facilitating the release and subsequent nuclear translocation of Nrf2, establishing a positive feedback loop.^134^, ^135^, ^136^ Bartolini et al concluded that the physical interaction between p62 and Keap1 modulated the U-shaped response of the Nrf2 transcription factor, thereby influencing its coordination with autophagy^137^ (Fig. 4).

Several studies have demonstrated that certain plant-derived compounds, traditional Chinese medicine monomers, and metabolic enzymes can influence the autophagy of CRC cells by modulating Nrf2 activity, thereby impacting the progression of CRC. Cannabidiol, a phytocannabinoid derived from *Cannabis sativa* L., demonstrates anti-tumor properties. Specifically, cannabidiol induces a p53-dependent overproduction of ROS, thereby activating p53-dependent caspase-8/9/3 pathways, stimulating the Keap1–Nrf2 signaling pathway, and triggering macroautophagy in HCT116 p53 wild-type cells. Hsp70 exhibits a complex interaction with p53, and the inhibition of Hsp70 can redirect cannabidiol-induced autophagy towards caspase-8/9-mediated apoptosis.^138^ The study conducted by Pandey et al demonstrated that fisetin, a dietary flavanol, could inhibit autophagy in CRC cells, thereby inducing apoptosis through mechanisms involving elevated ROS levels and a reduction in mitochondrial membrane potential. Additionally, the fisetin-mediated reduction in nuclear Nrf2 levels is identified as a critical target for its therapeutic effects.^139^ Hsu et al demonstrated that l-selenocystine (SeC) diminished the expression of Nrf2 and proteins associated with the autophagy pathway via the p62–Nrf2–ARE axis. This selective inhibition targets CRC cells with sustained Nrf2 activation, ultimately inducing cell death.^140^ The small leucine zipper protein (sLZIP), an isoform of LZIP, exhibits elevated expression in certain tumor progressions. In nutrient-deficient conditions, sLZIP is up-regulated, promoting LC3 transcription and initiating autophagy. This autophagy modulates the Keap1–Nrf2–NQO1 pathway, maintaining redox homeostasis in CRC cells under metabolic stress.^141^ Glutaminase (GLS1), crucial for glutamine metabolism, is highly expressed in various cancers. Liu et al discovered that depleting *GLS1* in CRC cells raised ROS levels and inhibited Nrf2 activation via an autophagy pathway involving LC3 and p62, thus hindering CRC cell proliferation and migration.^142^ Tributyltin (IV) ferulate (TBT-F), a novel organotin derivative of ferulic acid, has been shown to stimulate the production of ROS, leading to endoplasmic reticulum stress and autophagy in CRC cells, functioning as a pro-oxidant agent. Celesia’s study demonstrated that TBT-F activated the Nrf2-mediated antioxidant response and that Nrf2 deficiency significantly enhanced the anti-cancer effects of TBT-F in CRC.^143^ Besides, Zhou et al demonstrated that oxaliplatin treatment induced the up-regulation of thioredoxin domain-containing protein 9 (TXNDC9) and promoted autophagy in CRC cells. Additionally, TXNDC9 was found to decrease the sensitivity of HT29 cells to oxaliplatin and modulate the autophagic response of these cells to oxaliplatin treatment by regulating the expression of Nrf2.^144^ In a study examining the impact of ethanol (EtOH) on CRC progression, Cernigliaro et al demonstrated that high doses of EtOH induced autophagy and activated the Nrf2 expression in CRC cells. Furthermore, EtOH was found to promote a prosurvival effect in CRC cells by triggering autophagy in response to oxidative stress. Collectively, these findings suggest that EtOH facilitates the survival of CRC cells, with Nrf2 activation providing a protective mechanism against EtOH-induced oxidative stress.^145^ However, the findings of Enkhbat et al indicated that epigallocatechin-3-gallate (EGCG), a known Nrf2 inducer, enhanced the sensitivity of CRC cells to radiation by promoting the nuclear translocation of Nrf2 and up-regulating autophagy-related genes, thereby contributing to the suppression of CRC cells’ viability^146^ (Fig. 4).

Furthermore, elevated levels of ROS can induce a stress response that regulates autophagy via the activation of Nrf2. Autophagy plays a crucial role in the sensitivity of CRC cells to docosahexaenoic acid (DHA), suggesting that this fatty acid may have potential therapeutic effects against CRC cells exhibiting low levels of autophagy.^147^ In mice with colitis-associated colon carcinogenesis, the expression of Nrf2, NQO1, and HO-1 is reduced. Melatonin treatment inhibits autophagy by reducing inflammation and oxidative stress, which increases p62 levels and activates Nrf2, thereby boosting NQO1 and HO-1 expression. Melatonin interacts with autophagy and Nrf2 pathways to slow colitis-associated colon carcinogenesis progression. Thus, melatonin treatment mitigates colitis-associated colon carcinogenesis progression in mice by modulating autophagy and Nrf2 signaling.^148^ In summary, targeting Nrf2 activity and autophagy presents a promising therapeutic approach for CRC. Table 4 and Figure 4 provide summaries of the primary references discussed in this section.

**Table 4:** Effects of Nrf2 activity on the autophagic properties of CRC cells.

Cell/Animal models	Nrf2 manipulation	Autophagy-related indicators	Effects	Refs
HCT116 p53wt, LS174T p53wt, HCT116 p53^−/−^, SW480 p53mut	↑, Pharmacologic (CBD)	p62, LC3B	Inhibit proliferation	138
SW480	↓, Pharmacologic (Fisetin)	LC3-II, Beclin-1	Inhibit survival	139
WiDr, C_2_BBe_1_	↓, Pharmacologic (SeC)	ULK1	Inhibit survival	140
HCT116	↑, Genetic (sLZIP-OE)	LC3	Promote survival	141
DLD-1, SW480	↓, Genetic (GLS1 shRNA)	LC3B	Inhibit proliferation; Inhibit invasion	142
HCT116	↑, Pharmacologic (TBT-F)	LC3-II, p62	Inhibit proliferation	143
HT29	↓, Genetic (TXNDC9 shRNA)	LC3-I/II, p62	Reduce drug resistance	144
HCT116, HT29, Caco-2	↑, Pharmacologic (EtOH)	LC3, Beclin-1, ATG7, p62	Promote survival	145
HCT116	↑, Pharmacologic (EGCG) + RT	LC3	Inhibit proliferation	146
SW620, Caco-2	↑, Pharmacologic (DHA)	LC3B-II, SQSTM1, ATG12, ATG14	Promote drug resistance	147
Male Swiss Albino mice	↑, Pharmacologic (MEL)	Beclin-1, LC3-II/LC3-I, p62	Inhibit proliferation	148

ATG, autophagy-related genes; CBD, cannabidiol; CRC, colorectal cancer; DHA, docosahexaenoic acid; EGCG, Epigallocatechin-3-gallate; EtOH, ethanol; GLS1, glutaminase; LC3, microtubule-associated protein 1A/1B-light chain 3; MEL, melatonin; Nrf2, Nuclear factor erythroid 2-related factor 2; OE, over expression; RT, radiotherapy; SeC, l-selenocystine; sLZIP, small leucine zipper protein; TBT-F, Tributyltin (IV) ferulate; TXNDC9, thioredoxin domain-containing protein 9; “↑” or “↓”mean Nrf2 activation and inhibition, respectively.

The dual role of Nrf2 in autophagy, oscillating between protective and cytotoxic effects, remains mechanistically unclear due to inconsistent autophagic flux measurements, often relying on LC3/p62 rather than lysosomal inhibition assays. The tumor microenvironment is another neglected area; while cancer-associated fibroblast-secreted transforming growth factor-beta (TGF-β) activates Nrf2 to enhance protective autophagy, the influence of myeloid cell-derived ROS on this pathway is not understood. Methodological limitations remain, as most studies assess autophagy through LC3/p62 expression instead of functional assays like autophagic flux tracking, causing misinterpretations of autophagy status. To advance translational progress, it is essential to validate Nrf2-autophagy targeting in patient-derived organoids and clinical trials. This should emphasize biomarkers like p62 accumulation or lysosomal activity to predict responses and prevent off-target effects from autophagy inhibition.

### Nrf2 and other types of CRC cell death programs

The activity of Nrf2 is critical in determining the sensitivity of CRC cells to chemotherapy. The GSDME-N domain is a significant contributor to the promotion of pyroptosis. Silencing *Nrf2* increased GSDME-N expression in oxaliplatin-treated CRC cells due to higher ROS levels. These findings indicated that targeted modulation of Nrf2 activity may enhance chemotherapy sensitivity in CRC cells by inducing pyroptosis through ROS accumulation.^149^ Activation of Nrf2 induces the expression of the downstream antioxidant gene HO-1 in HUVECs, which subsequently influences the expression of key pyroptosis-related proteins, NLRP3 and caspase-1.^150^ A similar mechanism was observed in CRC cells, where GRh3, a traditional Chinese medicine, was found by Wu et al to inhibit Nrf2 nuclear translocation via the Stat3/p53/Nrf2 axis. This action suppresses HO-1 expression, inducing pyroptosis and exerting an anti-cancer effect.^109^

### Novel therapeutic strategies targeting Nrf2 and ROS in CRC

Modulating the Nrf2 pathway is a promising strategy for treating CRC due to its key role in managing oxidative stress and various cell death programs. Recent evidence indicates that targeting Nrf2, either directly or via its interaction with ROS, may improve the effectiveness of existing therapeutic interventions. This section provides a comprehensive overview of the current research landscape on Nrf2-targeting strategies, encompassing small molecule modulators, combination therapies, and prospective future directions.

### Small molecule modulators of Nrf2

Small molecule modulators of Nrf2 are attracting interest for their potential to affect ROS-mediated PCD in CRC. These compounds are mainly divided into Nrf2 activators and inhibitors, each with unique mechanisms and therapeutic effects. Brusatol and trigonelline can inhibit Nrf2 activity, weakening antioxidant defenses and making cancer cells more susceptible to ROS-mediated ferroptosis and apoptosis. For instance, brusatol boosted sorafenib’s effectiveness in liver cancer by promoting ferroptosis through Nrf2 and MT-1G suppression.^98^ NaB has been demonstrated to suppress the expression of Nrf2 and its downstream antioxidant gene *SLC7A11*, thereby inducing ferroptosis in CRC cells. *In vivo* studies indicated that NaB attenuated the progression of CRC in models of colitis-associated cancer by facilitating ferroptosis.^102^

Although Nrf2 activators are predominantly recognized for their cytoprotective properties, certain compounds can achieve therapeutic outcomes through the modulation of ROS and PCD. For example, EGCG, a prominent Nrf2 inducer, augmented the sensitivity of CRC cells to radiation by facilitating autophagy-dependent cell death.^146^ Curcumin triggered the activation of Nrf2 and its downstream antioxidant genes, subsequently inducing apoptosis in CRC cells via a ROS/Nrf2/miR-34a/b/c signaling pathway. This underscores the intricate role of Nrf2 in the pathophysiology of CRC.^53^ Developing selective Nrf2 modulators is challenging, as existing compounds often affect other pathways. More research is needed to explore agents that specifically target the Nrf2–Keap1 interaction without affecting other cellular functions.

### Combination therapies

Combination therapy emerges as a promising strategy to augment ROS-mediated cell death in CRC by integrating Nrf2 modulators with conventional chemotherapeutic agents or innovative therapeutic approaches. Table 5 summarizes the combination therapies targeting Nrf2-mediated PCD in CRC.

**Table 5:** Combination strategies targeting Nrf2 and PCD in CRC.

Combination therapy	Nrf2 activity	PCD mode	In vivo/vitro efficacy	Key caveats	Refs
PXA and CDDP	Inhibition	Apoptosis, Ferroptosis	Reduce tumor burden (HCT116 xenografts)	CDDP causes nephrotoxicity at high doses	151
MH and 5-FU	Inhibition	Apoptosis	Inhibit proliferation and migration (HCT116 and LoVo)	5-FU is toxic to surrounding normal cells	79
Cetuximab and RSL3	Inhibition	Ferroptosis	Inhibit viability and proliferation (HCT116 and DLD-1)	Cetuximab is clinically limited in KRAS-mutant CRC	104
EGCG and RT	Activation	Apoptosis, Ferroptosis	Inhibit proliferation (HCT116)	Lack of *in vivo* experiments	146
TFERL (pretreatment) and radiation	Activation	Ferroptosis (tumor), Cytoprotection (normal)	Reduce radiation enteritis (C57BL/6 mice); Inhibit proliferation (HT29 and SW620)	Active components of TFERL and its oral bioavailability remain unknown	110

CDDP, cisplatin; CRC, colorectal cancer; EGCG, epigallocatechin-3-gallate; MH, manuka honey; PCD, programmed cell death; PXA, penexanthone A; RSL3, RAS-selective lethal 3; RT, Radiotherapy; TFERL, Total flavonoid of Engelhardia roxburghiana Wall. Leaves.

### Nrf2 inhibitors and chemotherapeutic agents

Combining Nrf2 inhibitors with chemotherapy drugs like oxaliplatin, cisplatin, or 5-fluorouracil effectively boosts ROS-mediated cell death. For instance, penexanthone A, a potent Nrf2 inhibitor, disrupted redox balance and triggered ferroptosis, making CRC cells more sensitive to cisplatin-induced ferroptosis.^151^ Manuka honey inhibited Nrf2 expression, thereby synergistically enhancing the chemotherapeutic efficacy of 5-fluorouracil in CRC cells through the induction of ROS and the promotion of apoptosis.^79^

### Nrf2 inhibitors and ferroptosis inducers

The combination of Nrf2 inhibitors with ferroptosis-inducing agents, such as RSL3 and erastin, has exhibited significant anti-cancer efficacy in CRC. Notably, cetuximab, an EGFR inhibitor, facilitated ferroptosis in KRAS mutant CRC cells by suppressing the Nrf2/HO-1 signaling pathway.^104^

### Nrf2 activators and radiation

The combination of Nrf2 activators, such as EGCG, with radiation therapy augments therapeutic efficacy in CRC. This was achieved by promoting Nrf2 nuclear translocation, which enhanced antioxidative responses, while simultaneously inducing autophagic cell death.^146^ Furthermore, TFERL treatment had the potential to enhance the radiosensitivity of CRC cells while simultaneously alleviating radiation-induced intestinal injury through the activation of the Nrf2 pathway.^110^

Combination therapies not only improve the effectiveness of standard treatments but also target resistance mechanisms, including Nrf2-mediated chemoresistance. Future clinical trials must investigate the safety and efficacy of these combinations in CRC patients.

### Translational feasibility and biomarkers for Nrf2-targeted therapy

Developing Nrf2-targeted therapies is challenging due to tissue specificity and off-target effects. The therapeutic window is complicated by Nrf2’s dual role: activating Nrf2 protects normal tissues but may promote tumor resistance, while inhibiting Nrf2 increases tumor sensitivity but can damage normal tissues. Small-molecule Nrf2 inhibitors, like brusatol, can make CRC cells more sensitive to chemotherapy by reducing antioxidant defenses, but their non-selective nature may cause oxidative stress in normal tissues, such as liver damage at high doses.^152^ EGFR-targeted nanoparticles offer a promising way to reduce systemic toxicity and increase drug concentration in tumors.^153^ For Nrf2 activators, clinical utility is limited by potential tumor-promoting effects in early-stage CRC, emphasizing the need for patient stratification by Nrf2/Keap1 genetic status.^154^

Clinical trial progress for Nrf2 modulators in CRC is evolving, with preliminary evidence supporting combinatorial approaches. A phase I trial showed that the glutaminase inhibitor CB-839 combined with capecitabine is well-tolerated in advanced solid tumors and may improve progression-free survival in PIK3CA-mutant CRC patients.^155^ Mechanistically, CB-839 induces ROS accumulation and Nrf2 nuclear translocation, up-regulating uridine phosphorylase 1 (UPP1) to enhance 5-fluorouracil activation.^155^ A phase II trial of sorafenib with stereotactic body radiotherapy in CRC liver metastasis showed a 55% response rate in patients with high SLC7A11 expression, extending PFS compared with stereotactic body radiotherapy alone.^153^ Notably, no phase III trials of Nrf2 modulators for CRC have been completed, highlighting the need for larger cohorts to confirm efficacy.

Careful monitoring of Nrf2 modulation side effects is essential in clinical settings, as Nrf2 inhibitors can worsen intestinal toxicity in patients undergoing radiation therapy by weakening epithelial defense against radiation-induced ferroptosis.^153^ Nrf2 inhibitors such as metformin are generally safe for diabetics but may lead to vitamin B12 deficiency, necessitating supplementation in CRC chemoprevention.^80^^,^^156^ Combining Nrf2 inhibitors and ferroptosis inducers, like sorafenib, with stereotactic body radiotherapy generally leads to mild blood and gastrointestinal side effects, with severe cases in less than 15% of patients, indicating acceptable tolerability.^153^

### Future directions

Targeting the Nrf2 pathway, directly or via ROS interaction, is a promising strategy for CRC therapy. Small molecule Nrf2 modulators can regulate ROS-mediated PCD, making CRC cells more susceptible to apoptosis, ferroptosis, and autophagy. Combining Nrf2 inhibitors with chemotherapy or new treatments could improve therapy outcomes. Recent research mainly examines Nrf2’s involvement in apoptosis, ferroptosis, and autophagy, with limited evidence suggesting its impact on pyroptosis in CRC. Inhibiting Nrf2 boosted pyroptosis in CRC cells by raising ROS levels and GSDME-N expression.^149^ More studies are required to understand how Nrf2 regulates these less-explored PCD pathways and to evaluate their potential as therapeutic targets in CRC.

## Conclusion and perspectives

Nrf2 is a critical transcription factor that manages cellular adaptation to oxidative stress. A growing number of studies have demonstrated that Nrf2 is instrumental in the initiation and progression of cancer, particularly in CRC cells. In these cells, Nrf2 influences the cell death process by modulating ROS levels. As a pivotal transcription factor, Nrf2 regulates the cellular antioxidant response, thereby impacting cell survival and death. The Nrf2 signaling pathway plays a dual role in cancer cell apoptosis by protecting against oxidative stress and promoting cell survival through cytoprotective gene expression. In many cancers, Nrf2 activation increases resistance to apoptosis, enabling cancer cells to survive in environments with high levels of ROS and other stressors.^157^ In CRC cells, the activation of Nrf2 can promote apoptosis by modulating the NF-κB signaling pathway and p53, while inhibition of Nrf2 activity can induce apoptosis by affecting the PI3K–AKT pathway, which is closely associated with ROS accumulation.^74^^,^^78^^,^^158^ Furthermore, the accumulation of mitochondrial ROS can facilitate apoptosis in CRC cells by activating the MAPKs/p53-mediated caspase-dependent signaling pathway.^159^

Ferroptosis represents a form of cell death that is contingent upon the presence of iron and ROS. Studies have demonstrated that Nrf2 can inhibit ferroptosis in CRC cells by up-regulating the expression of SLC7A11, enhancing the synthesis of GSH, and suppressing lipid peroxidation.^101^^,^^105^ Additionally, Nrf2 might influence ferroptosis sensitivity by directly regulating the genes for SLC7A11 and FSP1, the key inhibitors of ferroptosis.^103^^,^^105^ In the investigation of Nrf2’s role in oxidative damage within intestinal epithelial cells, it has been demonstrated that the activation of Nrf2 can mitigate ROS levels by up-regulating the expression of antioxidant enzymes, thus inhibiting the excessive activation of mitophagy.^17^ Furthermore, Nrf2 influences ROS production by modulating mitochondrial function and energy metabolism, which subsequently impacts the autophagy process.^139^^,^^160^ In CRC cells, elevated ROS levels typically induce autophagy, with ROS accumulation being concomitant with the activation of the Nrf2 signaling pathway. Consequently, the targeted inhibition of Nrf2 activity has the potential to enhance autophagy in CRC cells, offering a novel therapeutic approach for CRC. Nonetheless, there is a paucity of research examining the role of Nrf2 in the regulation of ROS within other cell death modalities, such as pyroptosis and necroptosis, in CRC cells.

Notably, the CRC tumor microenvironment and non-epithelial components play a critical role in modulating Nrf2-mediated regulation of PCD. Inflammatory myeloid cells release ROS that temporarily activate Nrf2 in colonocytes, protecting them from apoptosis. However, prolonged ROS exposure leads to constant Nrf2 activation, contributing to chemoresistance.^161^ Hypoxia triggers HIF-2α, which increases lipid and iron regulation in CRC cells, making them prone to ferroptosis, but also promotes ROS buildup, enhancing cell death through iron-dependent pathways. Inhibiting HIF-2α lowers ROS levels and reduces cancer cell resistance to oxidative stress.^94^ In tumor-associated macrophages, Nrf2 activation resists reprogramming by interferon gamma (IFN-γ) and anti-CD40 antibodies, weakening their tumor-killing ability and aiding CRC progression.^162^ Additionally, Nrf2 activity in non-epithelial cells, like cancer-associated fibroblasts and myeloid cells, influences PCD by managing antioxidant defenses and ROS production. These findings underscore the necessity of considering tumor microenvironment crosstalk when developing Nrf2-targeted therapies for CRC.

In conclusion, the Nrf2 signaling pathway exhibits a multifaceted role in the progression of CRC, functioning both as a protector against oxidative damage and, under certain conditions, as a potential facilitator of cell death. There is a need for more comprehensive research to elucidate the effects of Nrf2 on CRC cell death programs, utilizing both animal models and clinical studies. A thorough understanding of these mechanisms is essential for the development of targeted therapies aimed at modulating this pathway for the treatment of CRC.

## CRediT authorship contribution statement

**Yichen Yu:** Writing – original draft, Investigation. **Bin Ma:** Writing – original draft, Investigation. **Yue Liu:** Writing – original draft. **Ziyi Wang:** Writing – review & editing. **Jian Chen:** Writing – review & editing. **Xiaoxi Li:** Writing – review & editing. **Shulan Sun:** Writing – review & editing, Writing – original draft, Funding acquisition. **Simeng Bao:** Writing – review & editing, Writing – original draft, Supervision, Funding acquisition, Conceptualization.

## Funding

This research was funded by the Liaoning Provincial 10.13039/501100008990Department of Science and Technology Doctoral Launch (China) (No. 2024-BS-317 to Simeng Bao), the 10.13039/501100001809National Natural Science Foundation of China (No. 82204090 to Zhiyuan Liu), and the Outstanding 10.13039/100003536Youth Foundation of Liaoning, China (No. 2022-YQ-08 to Shulan Sun).

## Conflict of interests

The authors declared no conflict of interests.
